# Refinement Measure Using NMR for Subcutaneous Fat Mass Determination in Rats

**DOI:** 10.3390/ani16142210

**Published:** 2026-07-16

**Authors:** Marina Colom-Pellicer, Mònica Lores, Xavier Escoté, Ximena Terra, Raúl Beltrán-Debón, Esther Rodríguez-Gallego, Montserrat Pinent, Anna Ardévol

**Affiliations:** 1 MoBioFood Research Group, Department of Biochemistry and Biotechnology, Universitat Rovira i Virgili, 43007 Tarragona, Spain; monica.lores@urv.cat (M.L.); ximena.terra@urv.cat (X.T.); raul.beltran@urv.cat (R.B.-D.); esther.rodriguez@urv.cat (E.R.-G.); montserrat.pinent@urv.cat (M.P.); anna.ardevol@urv.cat (A.A.); 2 Technological Unit of Nutrition and Health, Eurecat, Centre Tecnològic de Catalunya, 43204 Reus, Spain; xavier.escote@urv.cat

**Keywords:** adiposity, NMR, refinement, rat, fat mass

## Abstract

White adipose tissue (WAT) is categorized as either subcutaneous or visceral fat. One technique used to measure adipose tissue is nuclear magnetic resonance (NMR), which involves placing an animal in a tube for six minutes. This procedure is stressful for rats. Therefore, we evaluated whether determining the weight of subcutaneous WAT in sacrificed rats via NMR is a valid approach. Rats fed a standard diet showed similar WAT measurements when alive and after being sacrificed. Moreover, three groups of rats were fed different diets; one group was fed a cafeteria diet. The weight of WAT in the carcasses measured via NMR (mainly subcutaneous WAT) and the weight of the fat depots, measured after sacrifice (visceral WAT), presented similar percentages of subcutaneous and visceral WAT among the three groups (with different levels of adiposity). In conclusion, this research is a proof of concept regarding the determination of subcutaneous WAT weight via NMR, regardless of adiposity.

## Simple Summary

Excess body fat is linked to many health problems, and researchers often study it in animals to better understand these risks. One way to measure body fat in rats is by using a technique that involves placing the animal inside a tube for a few minutes, which can cause stress. In this study, we sought to determine whether this method could also be used reliably after the animals have been sacrificed, reducing the need to stress them while alive. We compared fat measurements taken before and after sacrifice and found that they were very similar. We also tested rats fed different diets, including a high-fat “cafeteria-style” diet that increases body fat, and observed that the distribution of fat was consistent across methods. Overall, our results show that this technique can accurately measure body fat even after the animal has been sacrificed. This finding can help improve animal welfare in research by reducing stress while still providing reliable data for studies on obesity and health.

## 1. Introduction

Body fat is a relevant measure analyzed in several experimental studies, largely because the degree of adiposity in an individual is a health-related parameter [1]. However, the localization of adipose tissue, rather than the amount, has been found to be more relevant to health impacts [2]. The main difference lies in whether the fat accumulates in the abdominal cavity (visceral white adipose tissue) or under the skin (subcutaneous white adipose tissue) [3].

There are several methods for quantifying body composition, such as measuring tissue and fluid masses. Nuclear magnetic resonance (NMR), dual-energy X-ray absorptiometry (DXA), and magnetic resonance spectroscopy (MRI) are three common methods [4,5]. One of the advantages of NMR is that animals do not need to be anesthetized, since it is not necessary to standardize their position in order to obtain accurate scans, unlike DXA and MRI, where this step is required in order to produce images of fat mass distribution [6].

Although NMR is a non-invasive technique, one of the requirements is that live animals must be relatively immobilized in a tube for around 6 min, which may cause acute stress [5]. To avoid this issue, rats could be placed in the NMR tube after being sacrificed. During sacrifice, the organs and visceral fat are usually removed for subsequent analysis. Thus, the rats are left as carcasses with mainly subcutaneous fat remaining under the skin but no organs. The carcasses can then be fed into the NMR scanner to measure the remaining fat, which is mainly subcutaneous. This study was designed according to the 3Rs principle [7], primarily addressing the refinement issue by reducing the stress and suffering experienced by the animals when they are immobilized in an NMR tub for several minutes. We hypothesized that NMR methodology could be the optimal tool for measuring subcutaneous adipose tissue in sacrificed rats, thereby avoiding suffering at this stage and enabling refinement of the measurement of sWAT in a diet-induced obesity model.

## 2. Materials and Methods

### 2.1. Animal Experiment

Twenty-nine 10-week-old Wistar rats were obtained from Janvier (Castellar del Vallés, Spain). The animals were housed in pairs at 22 °C and 55% humidity with a 12 h light/dark cycle. They were fed ad libitum a standard chow diet consisting of 72.4% carbohydrates, 21.6% protein, and 6% fat (ENVIGO Teklad Global, Barcelona, Spain), along with tap water, for one week. The rats were then housed individually until the end of the experiment.

Four female rats were fed ad libitum for six weeks for the pilot study. At the end of the experiment, NMR was used to analyze the fat and lean mass of the living rats. The animals were then sacrificed via decapitation, after which their tissues were collected and weighed. The tissues collected were the liver, right kidney, spleen, thymus, brown adipose tissue (BAT), and mesenteric (mWAT), gonadal (gWAT), inguinal subcutaneous (isWAT), and retroperitoneal (rWAT) WAT. The stomach, caecum, and small and large intestines were weighed both with and without contents. Organs were removed based on a standard visceral depot excision protocol. Subsequently, the fat and lean mass of the sacrificed animals (empty carcasses), excluding the aforementioned organs, were determined via NMR, as were the respective weights of these carcasses.

The remaining twenty-five male rats were fed ad libitum for 9 weeks. Afterwards, they were fed special diets with different percentages of fat for 4 weeks. The standard-diet group (STD) (*n* = 8) was fed a diet consisting of 72.4% carbohydrates, 21.6% protein, and 6% fat (Teklad, Envigo++, Barcelona, Spain); the control-diet group (Control) (*n* = 8) was fed a diet consisting of 75.1% carbohydrates, 16.7% protein, and 8.2% fat (personalized diet Envigo++, Barcelona, Spain) (submitted manuscript); and the cafeteria-diet group (CAF) (*n* = 9) was fed a diet consisting of 51% carbohydrates, 14% protein, and 35% fat [8]. At the end of the experiment, the animals were sacrificed, and their tissues were collected and weighed as previously described. Fat and lean mass were determined via NMR in sacrificed rats (empty carcasses) after all visceral fat depots (mesenteric, gonadal, and retroperitoneal) and organs had been completely excised.

All procedures received approval from the Comissió d’Experimentació Animal de la Generalitat de Catalunya (code: 11817). We calculated the sample size, accounting for the fact that the male Wistar rats were fed three different special diets for four weeks (three groups). The standard deviation was 40 g; the difference between groups was 50 g; the statistical significance (α) was 0.05; and the power (1 − β) was 95%. The proper sample size was determined to be eight rats per group.

### 2.2. Nuclear Magnetic Resonance (NMR)

NMR measurements were performed using an EchoMRI-700TM device (Echo Medical Systems, LLC, Houston, TX, USA). Prior to each session, the NMR was calibrated using the calibration tubes provided. The protocol included triplicate measurements for each rat. In the case of dead rats, the bodyweight considered for measurement was that of the “empty carcasses”.

### 2.3. Data Analysis

Data analysis was performed using XLSTAT 2020.1 (Addinsoft, Barcelona, Spain), and graphics were prepared using GraphPad Prism 9 (GraphPad Software, San Diego, CA, USA). Data are presented as means ± SEMs. The Shapiro–Wilk test was performed to analyze the normality of the data (*p* > 0.05). One-way ANOVA followed by the Bonferroni post hoc test was used to identify differences between rats or between dietary groups. Statistical significance for differences was set to correspond to *p* values < 0.05.

## 3. Results & Discussion

**Figure 1:**
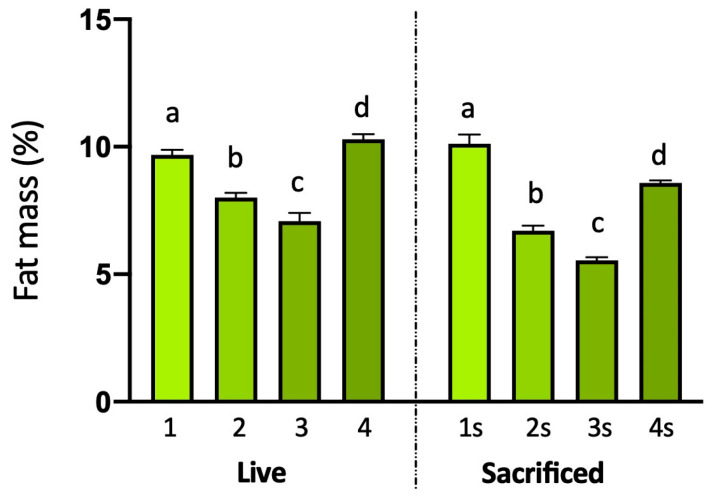
Fat mass percentage (%) in live and sacrificed rats determined by NMR (*n* = 4 female rats). Each bar represents three measurements of fat mass per live rat or sacrificed rat carcass after organ removal, as determined by NMR. In live rats, fat was divided by live bodyweight. In sacrificed rats, fat was divided by bodyweight after sacrifice and organ extraction (carcass). The letters indicate significant differences between each animal in the respective live or sacrificed rats determined using one-way ANOVA followed by the Bonferroni post hoc test (*p* < 0.0001).

To address the main objective of this study, we compared NMR measurements taken from the same animals while they were alive and from their respective carcasses once they had been sacrificed. Figure 1 shows the body fat percentage of each individual live and sacrificed rat, as determined by NMR. For the live animals, with a mean bodyweight of 283.8 ± 4.64 g, these data include all the adipose tissue. For the sacrificed rats, however, with a mean carcass weight of 200.2 ± 3.30 g, they include only subcutaneous adipose tissue.

The body fat percentage differed among the live rats but, interestingly, showed the same pattern in the measurements obtained from the sacrificed rats. Correlation analysis between the live and sacrificed animals produced a Pearson correlation value of 0.88 with a *p* value of 0.118, suggesting there is a strong correlation between both measurements, although the small number of rats prevented attainment of any statistically significant differences.

**Figure 2:**
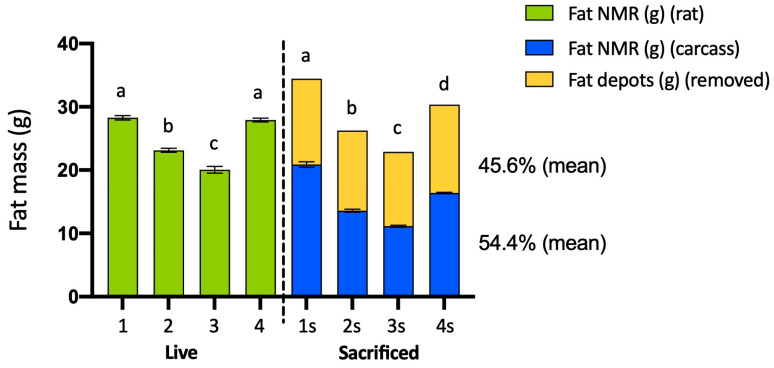
Total fat mass in living and sacrificed rats measured via NMR (green and blue), and fat depots weighed at sacrifice (orange) (*n* = 4 female rats). Each bar represents three measurements of fat mass per rat, except for fat depots, which correspond to a single measurement. Total fat was measured via NMR in live rats, while in sacrificed rats, it is the sum of the NMR measurement and the weight of fat depots removed at sacrifice. The letters indicate significant differences between each animal in the respective live or sacrificed rats determined using one-way ANOVA followed by the Bonferroni post hoc test (*p* < 0.0001).

Regardless of the low sample size, a complete comparison between the two situations (live and sacrificed) can be found in Figure 2. In the sacrificed animals, the weight of the removed fat pads has also been added, and the data are presented in grams of adipose tissue. The same trend in the differences between individual animals is also evident here. In these animals, subcutaneous WAT represents 54.4 ± 2.54% of the total fat (with blue indicating the sacrificed rats), while the extracted fat depots represent 45.6 ± 2.54% of the total fat measured (with orange corresponding to the sacrificed rats). Nevertheless, organ removal was carried out in accordance with the standard visceral depot excision protocol. It should be noted that NMR cannot differentiate between sWAT and any residual visceral fat.

**Figure 3:**
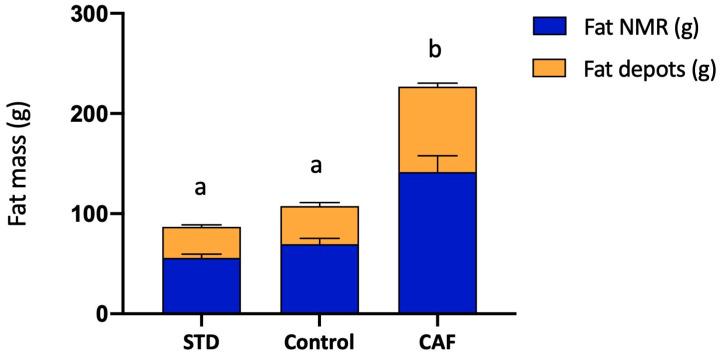
Total body fat (in grams) of sacrificed rats measured via NMR (blue) and fat depots weighed at sacrifice (orange). Fat from the carcass was measured via NMR, and fat depots removed at sacrifice were weighed. The letters indicate significant differences in fat weight among the groups (STD, standard diet (*n* = 8); control, control diet (*n* = 8); CAF, cafeteria diet (*n* = 9)) determined using one-way ANOVA followed by the Bonferroni post hoc test (*p* < 0.0001). The percentages of fat measured via NMR and the percentages of the fat depots weighted were calculated for each group.

To demonstrate the effectiveness of the refinement specifically for sWAT measurements in a diet-induced obesity model, we conducted an experiment involving rats that were fed different diets (STD, Control, and CAF). Statistically significant differences in bodyweight were only observed after the CAF diet treatment (STD: 586 ± 18.63 g; Control: 602 ± 17.38 g; CAF: 788 ± 29.06 g). Figure 3 shows the carcass weights and the weights of the removed fat depots after sacrifice and organ removal. After four weeks on a diet containing different percentages of fat, the changes in bodyweight and body fat were parallel, as shown in the Supplementary Material (Table S1). As expected, the animals fed the STD and control diets had significantly less fat than those fed the CAF diet. The subcutaneous fat detected in the carcass via NMR corresponded to 64.3 ± 1.32% (STD), 64.7 ± 0.68% (control), and 63.1 ± 2.11% (CAF) of the total fat content, while the extracted fat depots corresponded to 35.7 ± 1.32% (STD), 35.3 ± 0.68% (control), and 36.9 ± 2.11% (CAF). Lean mass also presented a similar profile, showing a greater quantity of lean mass in the animals fed the CAF diet in both the NMR and organ weight measurements, as shown in the Supplementary Material (Figure S1). The same profile was observed for total water detected via NMR: the group fed the CAF diet presented a significantly greater quantity of total water (STD: 313 ± 11.49 g; Control: 312 ± 9.02 g; CAF: 353 ± 8.80 g).

These results suggest that applying NMR to sacrificed and eviscerated animals is an efficient way to determine carcass adiposity. NMR measures the remaining fat content of the entire carcass rather than a single fat depot. This approach reduces operator-dependent variability associated with tissue dissection, improves reproducibility, and preserves carcasses for subsequent analysis. Moreover, NMR provides a rapid, accurate, and non-destructive method for assessing body adiposity. It enables reliable quantification of fat mass, lean mass, and water quantity without the need for chemical lipid extraction and, simultaneously, preserves carcasses for subsequent histological, biochemical, or molecular analyses.

However, depending on the aim of the study, it might be more accurate to use MRI, DXA, NMR imaging, or fat depot weight measurements. For instance, the presence of tumors in elderly rats can affect body composition measurements. MRI or DXA can overestimate lean mass in this case. This problem does not occur when fat pads are weighed, as tumors do not overestimate the amount of lean or fat mass. If the aim is to analyze the infiltration of fat into muscle or organs, DXA and MRI would be suitable methods. If the aim is to determine the weight of different fat pads, weighing them directly would be the most accurate method. Therefore, the most accurate method will depend on the aim of the study and the age and sex of the subjects [9].

Interestingly, females tend to store more fat in the subcutaneous depots than males, who tend to store more fat in visceral adipose tissue [10,11]. In addition, the sex dimorphism in subcutaneous fat is also reflected in the biochemical, immunological, and hormonal states [12,13]. However, in this study, females presented a lower percentage of subcutaneous fat than male rats. The females had approximately 54% subcutaneous fat (Figure 2), while the males had approximately 64% (Figure 3). Nevertheless, the small sample size of the female group could not provide conclusive data. A larger sample is needed in order to reach a definitive conclusion on sexual dimorphism.

## 4. Conclusions

This study provides preliminary evidence supporting the accuracy of determining subcutaneous fat mass in sacrificed rats using NMR. Furthermore, this methodology eliminates the stress the animals experience. Determining subcutaneous fat from carcasses via NMR represents a measure of refinement and appears to be a valid proof-of-concept that warrants further validation in larger studies.

## Data Availability

The original contributions presented in this study are included in the article/Supplementary Material. Further inquiries can be directed to the corresponding author.

## Supplementary Materials

The following supporting information can be downloaded at: https://www.mdpi.com/article/10.3390/ani16142210/s1. Figure S1. Lean body mass (in grams) of sacrificed rats measured by NMR (blue) and the organs removed except fat depots (orange). Table S1. Final body weight (g) and adiposity (%) in the experimental groups at the end of the study.

## References

[1] Gallagher D., Heymsfield S.B., Heo M., Jebb S.A., Murgatroyd P.R., Sakamoto Y. (2000). Healthy Percentage Body Fat Ranges: An Approach for Developing Guidelines Based on Body Mass Index. Am. J. Clin. Nutr..

[2] Emamat H., Jamshidi A., Farhadi A., Ghalandari H., Ghasemi M., Tangestani H. (2024). The Association between the Visceral to Subcutaneous Abdominal Fat Ratio and the Risk of Cardiovascular Diseases: A Systematic Review. BMC Public Health.

[3] Scudeler M.A., Morreale S., Doretto-Silva L., Petri G., dos Santos J.F.R., Nassis C., de T. Correa O.M., Veridiano J.M. (2022). Effects of Topiramate, Bupropion and Naltrexone Isolated or Combined on Subcutaneous Adipose Tissue in Obese Rats. Einstein.

[4] Borga M., West J., Bell J.D., Harvey N.C., Romu T., Heymsfield S.B., Dahlqvist Leinhard O. (2018). Advanced Body Composition Assessment: From Body Mass Index to Body Composition Profiling. J. Investig. Med..

[5] Lemos T., Gallagher D. (2017). Current Body Composition Measurement Techniques. Curr. Opin. Endocrinol. Diabetes Obes..

[6] Reho J.J., Nakagawa P., Mouradian G.C., Grobe C.C., Saravia F.L., Burnett C.M.L., Kwitek A.E., Kirby J.R., Segar J.L., Hodges M.R. (2022). Methods for the Comprehensive in Vivo Analysis of Energy Flux, Fluid Homeostasis, Blood Pressure, and Ventilatory Function in Rodents. Front. Physiol..

[7] Sneddon L.U., Halsey L.G., Bury N.R. (2017). Considering Aspects of the 3Rs Principles within Experimental Animal Biology. J. Exp. Biol..

[8] Colom-Pellicer M., Rodríguez R.M., Soliz-Rueda J.R., de Assis L.V.M., Navarro-Masip È., Quesada-Vázquez S., Escoté X., Oster H., Mulero M., Aragonès G. (2022). Proanthocyanidins Restore the Metabolic Diurnal Rhythm of Subcutaneous White Adipose Tissue According to Time-Of-Day Consumption. Nutrients.

[9] Castro-Rodríguez D.C., Ibáñez C.A., Uribe J., Menjivar M., de los Ángeles Granados-Silvestre M., Gerow K.G., Nathanielsz P.W., Zambrano E. (2020). Strengths and Validity of Three Methods for Assessing Rat Body Fat across the Life Course. Int. J. Obes..

[10] Palmer B.F., Clegg D.J. (2015). The Sexual Dimorphism of Obesity. Mol. Cell. Endocrinol..

[11] Staiano A.E., Katzmarzyk P.T. (2022). Visceral, Subcutaneous, and Total Fat Mass Accumulation in a Prospective Cohort of Adolescents. Am. J. Clin. Nutr..

[12] Vasconcelos R.P., Peixoto M.S., de Oliveira K.A., Ferreira A.C.F., Coelho-de-Souza A.N., Carvalho D.P., de Oliveira A.C., Fortunato R.S. (2019). Sex Differences in Subcutaneous Adipose Tissue Redox Homeostasis and Inflammation Markers in Control and High-Fat Diet Fed Rats. Appl. Physiol. Nutr. Metab..

[13] Many G.M., Sanford J.A., Sagendorf T.J., Hou Z., Nigro P., Whytock K.L., Amar D., Caputo T., Gay N.R., Gaul D.A. (2024). Sexual Dimorphism and the Multi-Omic Response to Exercise Training in Rat Subcutaneous White Adipose Tissue. Nat. Metab..

