# Supplementary material for: Refinement Measure Using NMR for Subcutaneous Fat Mass Determination in Rats
Source: Animals (Basel). 2026 Jul 16;16(14):2210. doi: 10.3390/ani16142210 (PMC13403409; doi:10.3390/ani16142210)
Supplement: Supplementary file 1 [file animals-16-02210-s001.zip › animals-4386926-supplementary.pdf]

SUPPLEMENTARY MATERIAL

Figure S1. Lean body mass (in grams) of sacrificed rats measured by NMR (blue) and the organs removed except fat depots (orange). *Lean from the carcass was measured by NMR and organs removed at sacrifice were weighed. Letters indicate significant differences between the groups (STD, standard diet; control, control diet; CAF, cafeteria diet) using one-way ANOVA followed by Bonferroni post hoc test (p=0.004).*

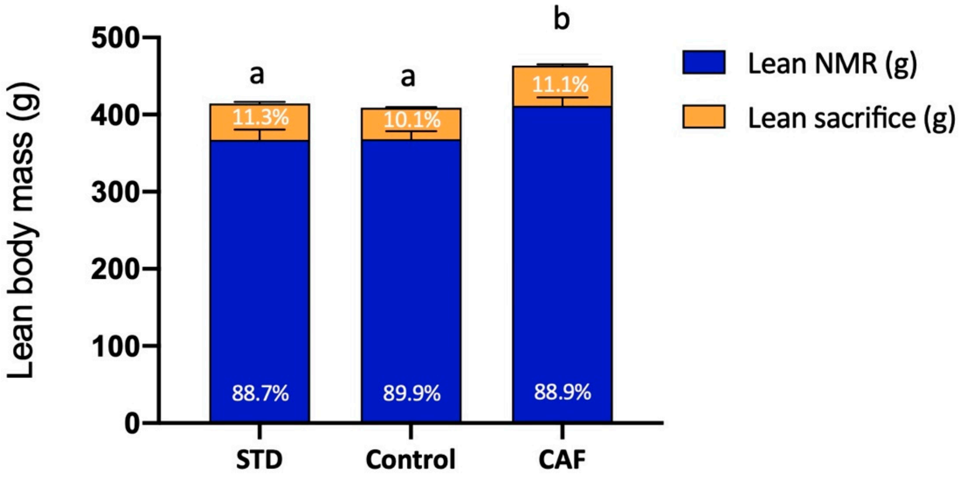

Table S1. *Final body weight (g) and adiposity (%) in the experimental groups at the end of the study. Data are presented as mean ± SEM. Different letters indicate statistically significant differences between groups, as determined by one-way ANOVA followed by Bonferroni's post hoc test (p < 0.0001).*

Variable	STD	Control	CAF	ANOVA (p)
**Body weight (g)**	586 ± 18.6 **a**	602 ± 17.4 **a**	807 ± 25.6 **b**	<0.0001
**Adiposity (%)**	14.9 ± 0.95 **a**	17.8 ± 1.39 **a**	29.1 ± 1.35 **b**	<0.0001
